# A Rare Presentation of Cardiac Lipoma as an Acute Coronary Syndrome: A Case Report and Review of Literature

**DOI:** 10.7759/cureus.15503

**Published:** 2021-06-07

**Authors:** Ahmed Younes, Soban Ahmad, Amman Yousaf, Constantin Bogdan Marcu

**Affiliations:** 1 Internal Medicine, East Carolina University, Greenville, USA; 2 Radiology, Hamad General Hospital, Doha, QAT; 3 Radiology, Services Hospital Lahore, Lahore, PAK; 4 Cardiovascular Sciences, East Carolina University, Greenville, USA

**Keywords:** acute coronary syndrome, mri, cardiac, lipoma, chest pain

## Abstract

Cardiac lipomas are rare benign cardiac tumors that are seldom symptomatic. We present a case of a 49-year-old female who presented with one week of substernal chest pain and uncontrolled hypertension. Initial workup showed left ventricular hypertrophy with non-specific intraventricular delay and T wave inversion in leads I and aVL on electrocardiogram (EKG), troponinemia, and elevated brain natriuretic peptide levels. A transthoracic echocardiogram showed mildly reduced left ventricular ejection fraction and severe segmental hypokinesis of the left ventricle. The patient was admitted to the hospital as a case of non-ST elevation myocardial infarction (NSTEMI), and appropriate treatment was commenced. The patient underwent an urgent coronary angiogram that showed no significant epicardial coronary artery disease. Subsequently, a gadolinium-enhanced cardiac MRI (CMR) was performed to rule out underlying structural abnormalities, which demonstrated a well-demarcated cardiac mass involving the left ventricular wall with characteristic features of cardiac lipoma. The patient had a favorable prognosis with conservative management, and she was discharged home in stable condition with a close follow-up for repeat CMR.

Although more studies are required, we suggest that cardiac MRI should be considered in patients with NSTEMI and non-revealing coronary angiography to rule out underlying cardiac tumors such as cardiac lipoma.

## Introduction

Cardiac lipomas are rare benign tumors and represent less than 10% of all the primary cardiac tumors [1]. With recent advances in diagnostic imaging, the incidence of cardiac lipomas is increasing. Cardiac lipomas are usually asymptomatic and are found incidentally in patients undergoing cardiac workup for other pathologies. There is no specific gender or age predilection reported in the literature [2]. When symptomatic, they can present with a myriad of symptoms ranging from mild angina to life-threatening arrhythmias depending on their location [3]. Cardiac lipomas can be visualized by echocardiography, but they are usually best characterized on cardiac magnetic resonance imaging (CMR) [4]. We report a rare case of cardiac lipoma presenting as non-ST elevation myocardial infarction (NSTEMI).

## Case presentation

A 49-year-old Caucasian female with a past medical history of hypertension, psoriasis, morbid obesity, and tobacco abuse presented to our hospital for evaluation of chest pain. The patient had intermittent chest pain of one-week duration, substernal, pressure-like, non-radiating, and 10/10 in severity. There were no aggravating or alleviating factors. The pain was associated with diaphoresis, vomiting, exertional dyspnea, and swelling of the lower extremities. She did not have any recent cold-like symptoms, fever, or cough suggesting viral myocarditis.

On examination, her vital signs were as follows: temperature of 36.7 degrees Celsius, blood pressure of 182/112 mmHg, heart rate of 92 beats/min, respiratory rate of 18 breaths/min, SpO_2_ of 99% on room air, and body mass index (BMI) of 42.5 kg/m^2^. The cardiopulmonary examination was otherwise unremarkable.

**Figure 1:**
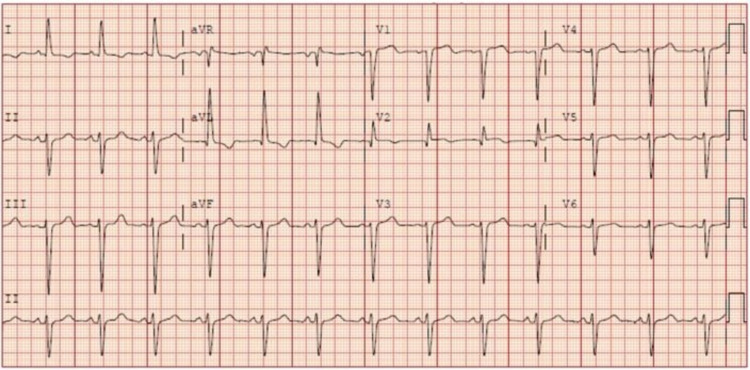
A 12-lead electrocardiogram showing left ventricular hypertrophy with non-specific intraventricular conduction delay and T wave inversion in leads I and aVL

Electrocardiogram (EKG) showed left ventricular hypertrophy by voltage criteria along with T-wave inversion in leads I and aVL (Figure 1).

Laboratory studies revealed elevated serum troponin I level of 1.91 ng/mL (reference range: ≤0.03 ng/mL) and brain natriuretic peptide level of 399 pg/mL (reference range: ≤100 pg/mL). The other blood investigations were within normal limits. A transthoracic echocardiogram showed reduced ejection fraction (45-50%) with severe hypokinesis of the mid-distal left ventricle. Other imaging, including a plain chest X-ray and a contrast CT pulmonary angiogram, did not reveal pulmonary infiltrates or pulmonary arterial embolism.

The patient was deemed to have NSTEMI based on the clinical presentation and elevated troponin level. She received aspirin and clopidogrel and was subsequently started on unfractionated heparin infusion as per the acute coronary syndrome (ACS) treatment protocol.

**Figure 2:**
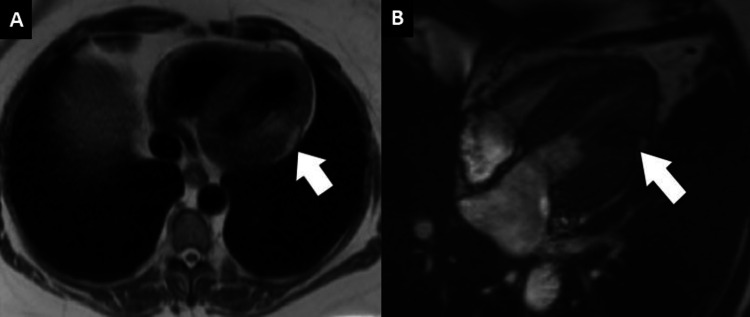
Gadolinium-enhanced cardiac MRI (A) T1-weighted sequence (axial view) showing regions of increased signal intensity (fat-containing) in the epicardial mid-lateral wall and base of the anterolateral papillary muscle. (B) T2-weighted sequence (axial view) showing decreased signal intensity in the same area.

The patient’s symptoms improved after the initiation of medical therapy. Given the regional wall motion abnormalities detected on echocardiography, the patient underwent elective coronary angiography, which showed no significant epicardial coronary artery stenoses. A gadolinium-enhanced cardiac MRI (CMR) was obtained for the diagnosis of MIOCA (myocardial infarct with open coronary arteries). CMR demonstrated interval normalization of regional and global left ventricular systolic function compared to echocardiography. However, a well-demarcated cardiac mass involving the epicardial, mid-lateral left ventricular wall, and the anterolateral papillary muscle base was detected. The mass signal characteristics on various CMR sequences and post-contrast were consistent with a cardiac lipoma (Figure 2).

**Figure 3:**
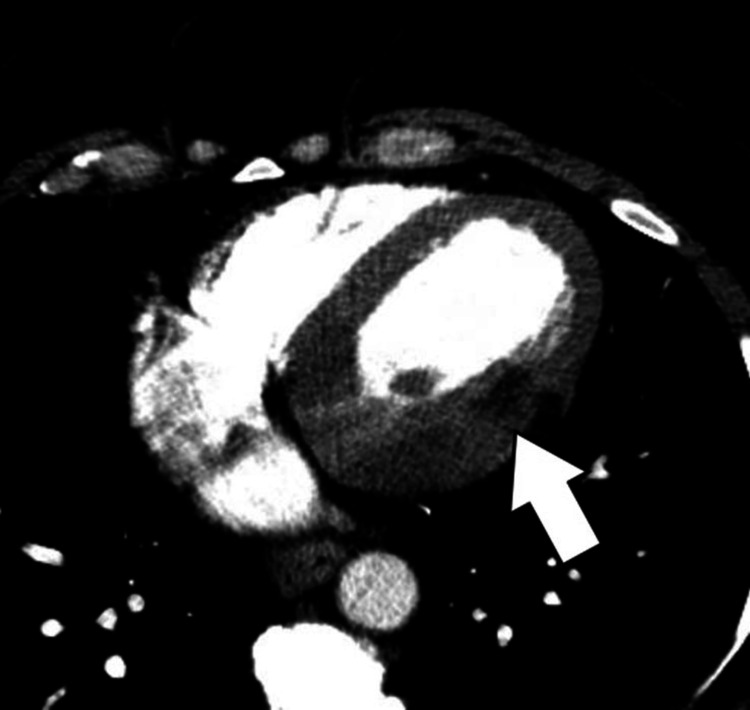
Contrast CT angiogram of the chest A selected axial view shows regions of fat attenuation (low HU) in the epicardial mid-lateral wall and base of the anterolateral papillary muscle, the same location as the cardiac magnetic resonance images (arrow).

Retrospective review of the initial contrast chest CT images correlated with the findings on CMR and showed regions of low-fat range attenuation in the same location (Figure 3).

The patient’s condition improved, and she was discharged home in a stable condition with a recommendation of an outpatient follow-up CMR in three months for surveillance. The patient continues to do well to date.

## Discussion

True population incidence of cardiac tumors is unknown as most of the literature data are derived from autoptic studies or echocardiographic registries [5]. Secondary tumors involving the heart either through metastasis or through direct local extension are far more common compared to primary cardiac tumors. Per literature review, more than 75% of the primary cardiac tumors are benign, 15% are malignant, and others, including paragangliomas and mesotheliomas, can be either benign or malignant [5]. The most common benign primary cardiac tumors in adults are cardiac myxomas followed by cardiac lipomas, representing about 8.4% of primary cardiac tumors [6].

Cardiac lipomas are well-encapsulated benign tumors of adipose tissue. Most lipomas originate from the subendocardium (50%), subepicardium (25%), and myocardium (25%). Typical sites include the left ventricle and right atrium [4]. Albeit benign and mostly asymptomatic, patients with cardiac lipoma can develop symptoms depending on the location and size of the tumor. Cardiac lipomas can cause compression of the coronary arteries leading to angina and myocardial infarction [3-5]. They can also cause embolic phenomenon and congestive heart failure depending on the size and location of the tumor mass, along with fatal arrhythmias and cardiac conduction abnormalities from myocardial infiltration [3,6]. Our patient also had left anterior fascicular block on EKG that might be secondary to the myocardial lipoma. Given the rapid recovery of regional wall motion abnormalities, it is likely that the patient had stress cardiomyopathy given interval normalization of left ventricular ejection fraction and clean coronary arteries.

Transthoracic echocardiography remains the initial diagnostic tool for most cardiac tumors, including lipomas, due to its easy accessibility, lack of radiation exposure, and non-invasive nature [7]. It may confirm the presence of a cardiac mass; however, due to its restricted field of view, dependence on the body habitus, and inability to characterize tissues, it provides limited information about the mass. Further evaluation is usually carried out by CMR and cardiac computed tomography (CCT). CMR is considered the imaging modality of choice due to better tissue characterization and the ability to differentiate between benign and malignant masses [8]. Cardiac lipomas have specific MRI characteristics that include high signal intensity on T1-weighted and T2-weighted images, signal suppression on fat-saturated images, and absent contrast enhancement [8,9].

There are no randomized controlled trials to guide treatment for cardiac lipomas. Due to their low prevalence and relatively benign nature, most asymptomatic patients are treated conservatively with close monitoring. Most experts recommend surgical excision in symptomatic patients, especially those having intractable cardiac arrhythmias, congestive heart failure, thrombo-embolic sequelae, or malignant features on imaging [6].

## Conclusions

Cardiac lipomas are rare tumors that can present with a wide variety of symptoms or can be asymptomatic. CMR is the imaging modality of choice, even though most of these tumors are discovered incidentally. A conservative approach with close surveillance is the cornerstone of management in the majority of the patients. Although more studies are required, we suggest that CMR should be considered in patients with NSTEMI and non-revealing coronary angiography to rule out underlying cardiac tumors such as cardiac lipoma.
